# Evolutionary ecology of Miocene hominoid primates in Southeast Asia

**DOI:** 10.1038/s41598-022-15574-z

**Published:** 2022-07-12

**Authors:** S. G. Habinger, O. Chavasseau, J.-J. Jaeger, Y. Chaimanee, A. N. Soe, C. Sein, H. Bocherens

**Affiliations:** 1 Department of Geosciences, University of Tübingen, Tübingen, Germany; 2 Laboratoire PALEVOPRIM, Université de Poitiers, Poitiers, France; 3 Mandalay University of Distance Education, Mandalay, Myanmar; 4 Department of Higher Education, Ministry of Higher Education, Nay Pyi Taw, Myanmar; 5 Senckenberg Centre for Human Evolution and Palaeoenvironment at the University of Tübingen, Tübingen, Germany

**Keywords:** Palaeoecology, Stable isotope analysis, Ecological modelling

## Abstract

The evolutionary history and palaeoecology of orangutans remains poorly understood until today. The restricted geographic distribution of extant *Pongo* indicates specific ecological needs. However, it is not clear whether these needs were shared by the great diversity of fossil pongines known from the Miocene to the Pleistocene. Here we show how niche modelling of stable carbon and oxygen isotope data of the carbonate fraction of dental enamel can be used to reconstruct the paleoecology of fossil and modern pongines and associated mammal communities. We focus on *Khoratpithecus ayeyarwadyensis*, a Late Miocene pongine from Myanmar and the sister clade to extant orangutans, and compare it to its associated mammal fauna and other fossil and extant pongines. The results are consistent with a vertical position high up in the canopy of a forested habitat with purely C_3_ vegetation for *K. ayeyarwadyensis* as well as the contemporaneous *Sivapithecus*. Although their positions in the modelled isotopic niche space look similar to the ecological niche occupied by modern *Pongo*, a comparison of the modelled niches within the pongine clade revealed possible differences in the use of microhabitats by the Miocene apes.

## Introduction

**Figure 1:**
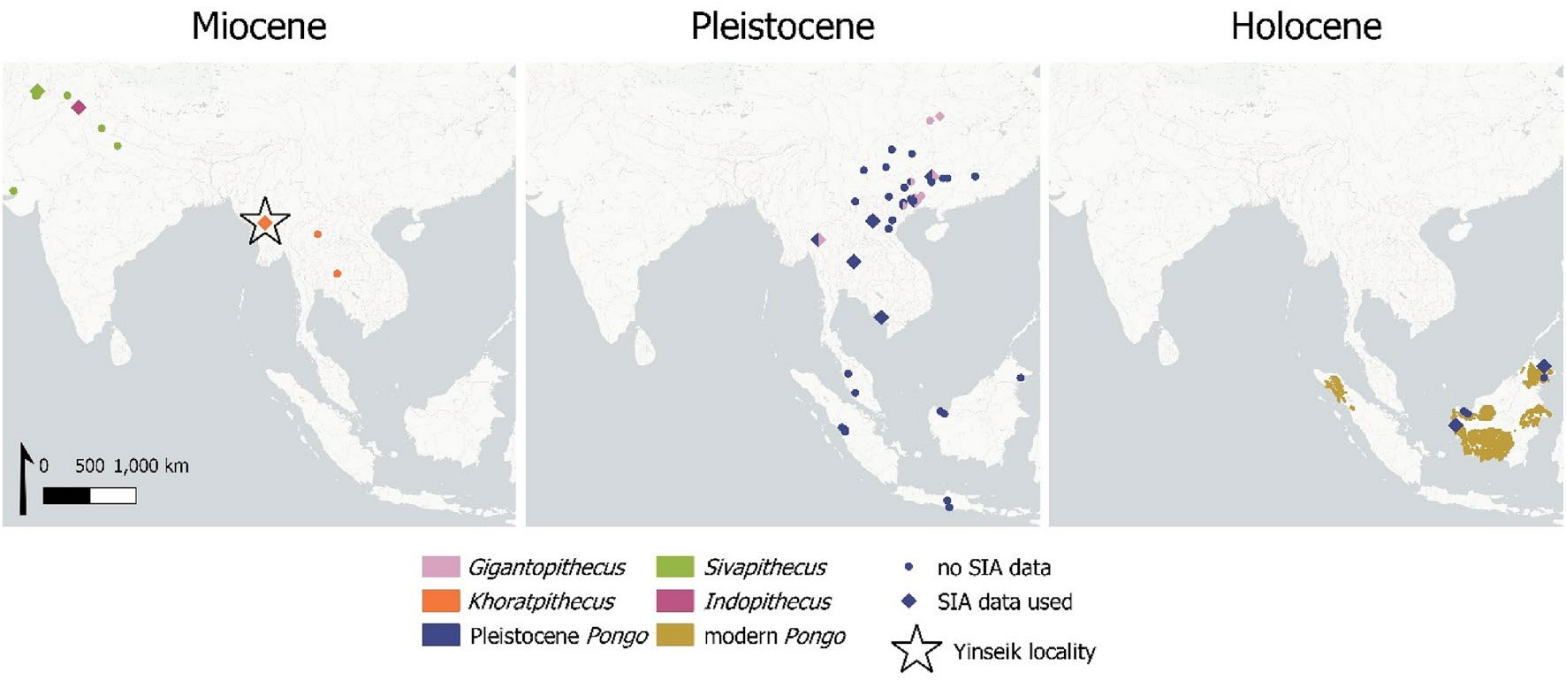
Location of fossil pongine localities from the Miocene to the Holocene in Southern Asia, Southeast Asia and Southern China including the present distribution of extant orangutans. The stable isotope data used in the study originates from the localities marked with a diamond (SIA = stable isotope analysis) and the Yinseik locality where *K. ayeyarwadyensis* was found is labelled with a star. A map version with labels for all the sites from which we used stable isotope data can be found in the Supplementary Information (Fig. SI 4). The map was created using QGIS 3.16.

Today, the only remaining genus of the Ponginae subfamily is the genus *Pongo*, including the three extant species of orangutans, whose distribution is highly restricted to forested areas of Borneo and Sumatra^1^ (Fig. 1). In contrast, paleontological data document a much greater diversity of pongine genera, with the earliest fossils recovered in Southeast Asia from the Chiang Muan Formation (Fm.) in Thailand and dating to 13–12.6 Ma^2,3^. Several different fossil pongine genera such as *Khoratpithecus* (Myanmar, Thailand), *Sivapithecus* (Pakistan, India, and Nepal), and *Indopithecus* (India) are known from the Miocene, as well as *Gigantopithecus* (China, Thailand, Vietnam) and early *Pongo* from the Pleistocene^4,5^. This high degree of diversification and endemism of the hominoid primates contrasts with the much more generalist character of the associated mammal fauna across different Southeast Asian sites^6–8^. The question now arises if this high pongine diversity also coincides with a diversity of subsistence strategies in this clade.

*Khoratpithecus* is known from isolated teeth, two mandibles and a maxilla^9–12^. This genus has been first allocated to the pongines based on shared derived dental and mandibular characters with *Pongo*^6,10^. It has been hypothesized that *Khoratpithecus* may represent the sister-group of *Pongo* based on the lack of an insertion for the anterior digastric muscle, a feature exclusively shared between these genera, and structural similarities of their mandibular symphyses^6,10^ (e.g., strong symphyseal inclination and weak upper transverse torus). More recently, the analysis of a maxilla of *Khoratpithecus* has revealed several additional pongine features for this genus in its upper dentition (e.g., externally rotated canines) and subnasal anatomy (e.g., strong overlap of the premaxilla relative to the maxilla, antero-posteriorly convex nasoalveolar clivus, subhorizontal incisive foramen)^11^. Phylogenetic analyses^5,13^ strengthen the hypothesis of a pongine clade including *Pongo* and large Asian Neogene hominoids (*Sivapithecus*, *Indopithecus*, *Gigantopithecus*, *Khoratpithecus*, *Ankarapithecus*, and irregularly *Lufengpithecus*). In the most recent phylogenetic analysis^5^, *Khoratpithecus ayeyarwadyensis* is regularly found as the sister-group of *Pongo*. Among the fossil genera of Asian hominoids, only the pongine status of *Lufengpithecus* is highly controversial according to investigations of its skull anatomy (in particular the orbital region)^14^ and further comparisons of its craniofacial features with other Asian Miocene hominoids^11^. This hominoid probably represents a stem hominid.

As the genus *Khoratpithecus* is hypothesized to be the sister-clade to *Pongo*^6^, investigating its paleoecology is crucial to understand the evolutionary history of modern orangutans. There are currently three different known species of *Khoratpithecus*. The Middle Miocene *K. chiangmuanensis*^9^ and the Late Miocene *K. piriyai*^6^ have been found in Thailand, but teeth from these two species were not available for sampling. However, one specimen of the Myanmar species *K. ayeyarwadyensis* could be sampled for stable isotope analysis. It is, together with its associated mammal fauna, the focus of our study. The fossil remains have been found in the Irrawaddy Fm. at a locality southeast of Magway near Yinseik village dating to the Late Miocene (~ 9.5 Ma)^12^. Hence, it will be referred to as Yinseik locality hereafter.

The important phylogenetic role of the genus *Khoratpithecus* as a sister clade of modern *Pongo* made us particularly interested in its palaeoecology and habitat. We wanted to characterize this habitat concerning its palaeoseasonality, vegetation structure, and the niche partitioning of the mammal fauna associated with *K. ayeyarwadyensis*. In a second step, we used the available stable isotopic data of fossil and extant pongines (Fig. 1) to assess if there is evidence for ecological continuity within the pongine clade, an important question to enhance our understanding of the evolutionary ecology of the Ponginae. The response of fossil pongines to climatic changes and forest fragmentation additionally can provide information for conservation efforts of orangutan populations today, which are continuously put under stress due to anthropogenic disturbances and habitat loss^15,16^. Similar conditions of continuing forest fragementation have been hypothesised to be the cause of the extinction of *Sivapithecus* during the Late Miocene^17^.

To answer these questions, we performed stable isotopic analysis (SIA), and used these data to reconstruct the niche partitioning in this habitat, as well as palaeoseasonality and to get a first direct indication of the ecology of this fossil pongine. Carbon and oxygen isotopes from the carbonate fraction of dental enamel that we analysed are commonly used as proxies for vegetation type^18,19^, and canopy density^20,21^; temperature, humidity^22^, as well as vertical stratification^23,24^ in primates, respectively. In this study, we will discuss *δ*^13^C values corrected for isotopic enrichment from diet to bioapatite and for changes in the isotopic composition of CO_2_. These corrections are discussed in detail in the Methods section. With these data, we modelled the core ecological niches of the different taxonomic groups and could characterize the niche partitioning in the Yinseik mammal fauna, quantify niche width and niche packing.

The modelled ecological niches form the basis for our comparison of the *Khoratpithecus* fauna with a younger locality from the Irrawaddy Fm, which did not yield any *Khoratpithecus* fossils^25^. This is a similar approach as the comparison of the *Sivapithecus* and the post-*Sivapithecus* fauna performed by Nelson^17^. This comparison will enhance our understanding of the paleoecology of *K. ayeyarwadyensis* and the changes happening in its habitat especially regarding the introduction of C_4_ plants and the general opening of the landscape. Ecological core niches were also modelled for other fossil and extant pongines. These niches were compared to one another to see, if they are consistent with the hypothesis of ecological continuity in the Ponginae.

## Material and methods

The fluvial deposits of the Irrawaddy Fm. represent one of the most important fossiliferous formations located in the Central Basin of Myanmar (Fig. 1). The hominoid-bearing locality of Yinseik from which the *K. ayeyarwadyensis* specimen and the associated mammal fauna analysed in this study originate is located close to the village Yinseik, east of the Irrawaddy River and about 20 km southeast of Magway (Magway Region). The section in the Yinseik area, which represents the base of the Irrawaddy Fm., is composed of about 100 m of fluviatile sediments with low dipping mostly constituted of poorly consolidated and coarse sandstones and hardened fine sandstones alternated with clayey sandstones and claystones. Oxidized conglomeratic layers also occur and are frequently fossiliferous. These deposits most probably represent a small lapse of time because they have yielded a homogeneous vertebrate fauna and have recorded a single normal magnetic polarity^12^. A 10.4–8.6 Ma age bracket (early Late Miocene) has been as inferred from biochronology. Two ages within this bracket, ~ 10 Ma and ~ 9 Ma, are compatible with the magnetostratigraphy^12^. There are great similarities in the composition of the associated mammal fauna of *K. ayeyarwadyensis*^7^ and of the contemporaneous faunas associated with *K. piriyai*^6^ and *Sivapithecus*^8^.

For this study, we sampled 44 teeth of various taxa, i.e. Rhinocerotidae (n = 9), Proboscidea (n = 8), Bovidae (n = 11), Suidae (n = 7), Giraffidae (n = 5), and one specimen of *K. ayeyarwadyensis* (MFI-K171) as well as one cervid and one anthracothere. The pongine specimen is a left hemi-mandible of K. ayeyarwadyensis, which is also the holotype of this species^12^. Sampling it for isotopic analysis was possible, as during preparation of the fossil a small enamel fragment of the m3 broke of, which was then analysed. We also included one modern bovid from the same area in our data set. Data from the Yinseik Equidae (n = 6) from the Yinseik locality has already been published by Jaeger et al.^12^.

Data from published studies with a similar scope and objective were used as reference (Table SI 3)^12,17,25–37^. These studies also analysed the carbonate fraction of dental enamel. However, the *Indopithecus* specimen was sampled using laser ablation instead of micro drilling. We use the normalized *δ*^13^C and *δ*^18^O values proposed by the authors^26^ to compare them to the other conventional CaCO_3_ data. However, especially the *δ*^18^O values might still be biased, due to the different methodologies. We complemented our data by the addition of Louys and Roberts^24^ data set on modern orangutans. Precise taxonomic information and specimen numbers of the whole data set used in this study are reported in Table SI 2.

### Sample preparation

The enamel was sampled using a micro dremel tool to retrieve 6–10 mg of powdered sample during fieldwork in Myanmar conducted in 2017. We took bulk samples of most of the teeth, where the drilling was done in a band along the whole growth axis of the tooth. Values represent the average isotopic composition over the mineralization time of the tooth during ontogeny. For some high-crowned teeth, it was possible to conduct intra-individual serial sampling (IRWD-9, IRWD-17, IRWD-24, IRWD-31, IRWD-42, and PND-M1). In these cases, multiple samples were drilled from bands perpendicular to the growth axis of the tooth providing a continuous record of isotopic variation during the mineralization time of this tooth.

The powdered samples were pretreated and the carbonate fraction of the dental enamel was analysed in the laboratory of the Department of Geosciences (Biogeology working group) at the University of Tübingen (Germany). They were let to react with 1.35 ml of a NaOCl solution at a concentration of 2.5% for 24 h to remove all the organic matter. After rinsing them with Milli-Q H_2_O the samples reacted with 1.35 ml of 1 M acetic acid buffered solution (CH_3_COOH) for 24 h to remove all exogenous carbonates. The method for the pretreatment of the samples followed the methodology described in^38–40^. When the reaction was completed, the samples were dried at 40 °C. With each set of samples, two internal modern enamel standards (Elephant SRM (SRM = secondary reference material), Hippo SRM) were processed following the same pretreatment protocol. The internal standards were complemented by two international (IAEA-603, NBS-18) and one internal (LM = Laaser Marmor SRM) pure carbonate standards. Pure carbonate standards were not subjected to any pretreatment. All of the standards were measured after every 15 samples in the IRMS (isotope ratio mass spectrometer).

2.5–3 mg or 0.1 mg of sample for enamel and pure carbonates respectively was then reacted with phosphoric acid (H_3_PO_4_) at a concentration of ~ 99%. The CO_2_ gas that resulted from this reaction was then analysed with the Elementar IsoPrime 100 IRMS 5 times over a 15-min time span. These repeated measurements were then used to monitor measurement precision by calculating the mean and the standard deviation for each sample^41^. Measurement uncertainty, as assessed using the standards and is reported for each sample in Table SI 2. The measured isotopic ratios were then calibrated relative to VPDB (Vienna Pee Dee Belemnite) using the two internal enamel standards. They are reported using the *δ*-notation (in per mill) whose calculation is based on the following formula^42,43^ where ^j^X is the heavier and ^i^X the lighter isotope.

1 $${\delta }^{j/i}X=\frac{{(}^{j}X{/}^{i}X{)}_{sample}}{{(}^{j}X{/}^{i}X{)}_{standard}}-1$$

We also report an estimation of the CaCO_3_ content of each sample (Table SI 2). With the Ion Vantage software, we calculated an estimated elemental composition based on sample weight, peak area and the internal LM SRM. The obtained CaCO_3_ values are then scaled up, until one of the international pure carbonate standards (IAEA-603, NBS-18) reaches 100%.

### Data correction

To enable the comparison of specimens from different species, sites, and time periods it was necessary to apply different corrections of the data, especially the *δ*^13^C values. All the *δ*^13^Capatite values were transformed to *δ*^13^Cdiet values. This was done using different enrichment factors (*ε*, in per mill) for different groups of animals. It is calculated using this formula^42,44^, where a stands for diet and b for apatite:

2 $${\varepsilon }^{13}{C}_{a-b}= {\alpha }^{13}{C}_{a-b}-1=\frac{{\delta }^{13}{C}_{a/standard}+1}{{\delta }^{13}{C}_{b/standard}+1}-1$$

*ε* is based on the isotopic fractionation factor (*⍺*), which is derived from the δ-values as defined in (1). Isotopic fractionation from diet to apatite is not explainable by a single kinetic or equilibrium process^42^. We account for this complexity by the use of the terms apparent isotopic fractionation factor (⍺*) and apparent enrichment factor (ε*) in the rest of this paper. Using Δ (*δ*^13^C_diet_ − *δ*^13^C_apatite_) decreases in accuracy when the isotopic differences among tissues are ≥ 10‰^45^, which is the reason why we decided to use *ε* instead.

We applied apparent enrichment factors based on the results of published studies of controlled feeding experiments of − 14‰ for large-bodied, ruminant herbivores^45,46^, − 11‰ for omnivores including suids^46,47^ and primates^48^.

For the comparison of animals from different time periods it is necessary to correct the *δ*^13^C values for changes of the *δ*^13^C_CO2_ values in the atmosphere caused by the Suess effect^49^. All values have been corrected to the pre-industrial values from 1850 of − 6.5‰^50^. The *δ*^13^C_CO2_ values of Miocene data points from sites that are older than 6 Ma were assumed to be − 6.1‰ and therefore a correction of 0.4‰ was applied to them^51^. Modern atmospheric *δ*^13^C_CO2_ values are − 8‰^52,53^, so all post 1930 samples were corrected by 1.5‰. Pre 1930 to 6 Ma old values are treated as equivalent to the pre-industrial ones^54^, and hence are not corrected. As the maximum age of the Chaingzauk fauna, from the other Miocene locality of the Irrawaddy Fm., is constrained to 6 Ma by its biostratigraphy^25^ no corrections were applied to the *δ*^13^C values from this site. The *ε* as well as the atmosphere correction used for each specimen are reported in Table SI 2 and Table SI 3 together with the calibrated data and corrected *δ*-values used in the analysis.

### Statistical analysis

For this study, we applied different statistical methods, which we want to describe in the following section. Given the small sample sizes in our data set as well as the presence of outliers and not normally distributed data we decided to use the non-parametric Wilcox rank sum test when testing for differences of the medians between two groups throughout this study. The tests were run in R using the wilcox.test() function (package stats version 4.1.2, one of the base packages in R).

We re-ran the linear regression on minimum *δ*^18^O values over time (Ma = million years ago) published by Nelson^55^ using the lm() function (package stats version 4.1.2, one of the base packages in R) and added that data from the Yinseik equids to the figure in order to compare them to the regression line.

The lowest *δ*^18^O value (− 5.7‰) measured in the serially sampled modern bovid specimen (PND-M1) looked like an anomaly during visual inspection. We therefore wanted to test if it was a statistical outlier. Before that, it was necessary to test if the assumption of the Grubbs’ test that the data are normally distributed was fulfilled. Therefore we conducted a Shapiro–Wilk test of normality using the shapiro.test() function (package stats version 4.1.2, one of the base packages in R). The results of this test for the *δ*^18^O value of PND-M1 show that the *p*-value is bigger than 0.05 (W = 0.94899, *p*-value = 0.4094), indicating that we do not reject the null hypothesis that the data follow a normal distribution. The Grubbs’ test for outliers was run in R using the grubbs.test(data, opposite = T) function (package outliers^56^ version 0.15) to test, if the minimum value of the data set was an outlier.

To be able to quantify and better compare the niche width and niche partitioning of the different mammal communities we applied isotopic niche modelling. Until now, isotopic niches based on the niche concept of Hutchinson^57,58^ have been mostly limited to dietary or trophic niches based on carbon and nitrogen isotopic composition of collagen^59^. As we analysed the carbonate fraction of the fossil dental enamel and therefore have data on two proxies reflecting more general ecological characteristics of an individuals’ habitat, we will model more general ecological niches for the pongines and the associated mammal fauna using the R package SIBER (version 2.1.6)^60^. Nelson and Hamilton already adopted a similar approach focusing on the dietary transition and ecological niche of early humans^61^. In this study we use standard ellipse area corrected for small sample sizes (SEAc) that corresponds to a confidence interval (CI) of 40%^60^. These calculations are based on a maximum likelihood estimation in a Bayesion framework. This framework is well suited for small sample sizes in general as it counteracts their effects on the statistical power of the analysis to a certain extent. However, it should be noted that increasing the sample size especially for the Miocene hominoids would lead to more robust results.

Statistical tests and modelling (linear models and Bayesian niche modelling) were conducted using R version 4.1.2 (2021-11-01) “Bird Hippie”. Most figures were generated using Excel (2016) except for the maps for which we used QGIS (version 3.16) and the visualisation of the models, which were created using the plot functions in R (packages ggplot2 version 3.3.5, and SIBER version 2.1.6). All figures were further modified using GIMP (version 2.10.18).

## Results and discussion

### Paleoseasonality estimations for Late Miocene of Myanmar

Intra-individual variation of *δ*^18^O values is routinely used to infer paleoseasonality. In regions with monsoonal climate, the temperature effect on these values is overwritten with the amount effect^22^. Therefore, the wet season is characterized in these regions by lower *δ*^18^O values and the dry season by higher ones.

**Figure 2:**
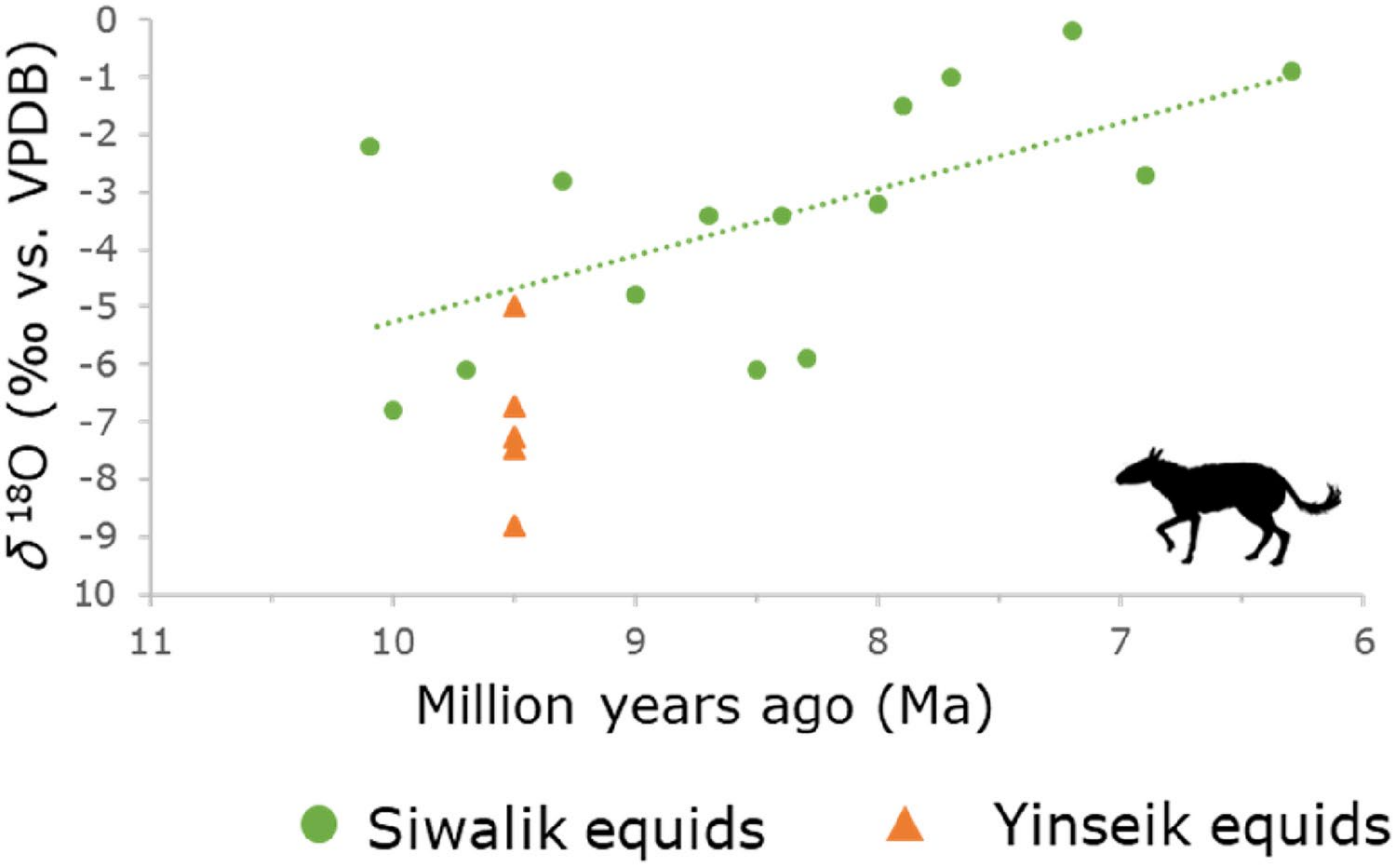
Development of the minimum δ^18^O values from dental enamel of hipparions from the Siwaliks^21^ over time. The linear regression line. (y = − 1.1527x + 6.2827; adjusted R^2^ = 0.3216; *p*-value = 0.01611) based on these values shows a trend towards more positive δ^18^O values towards the end of the Miocene corresponding to lower seasonal maxima of precipitation. Minimum δ^18^O values from the Yinseik specimens were added in orange. All of them plot below the regression line among the lowest minima from the Siwalik hipparions. Icon obtained via PhyloPic and in public domain.

Sun and collaborators interpreted the lower *δ*^18^O values from the Shuitangba locality (~ 6.2 Ma) from the Yunnan province in southwest China, in comparison to modern reference data, as an indication of a wetter climate during the Late Miocene than today^62^. The same pattern is also present in the hipparions from the Siwaliks of Pakistan^55^. The minimum *δ*^18^O values of the carbonate fraction of their dental enamel measured through the serial sampling of hypsodont teeth rise over time, which is consistent with a decreasing amount of seasonal precipitation (Fig. 2). For the Late Miocene, seasonal precipitation regime with a dry season of five to six months (similar to the monsoonal forests in southern China today) was inferred from intra-individual serial sampling of equid dental enamel^55^.

**Figure 3:**
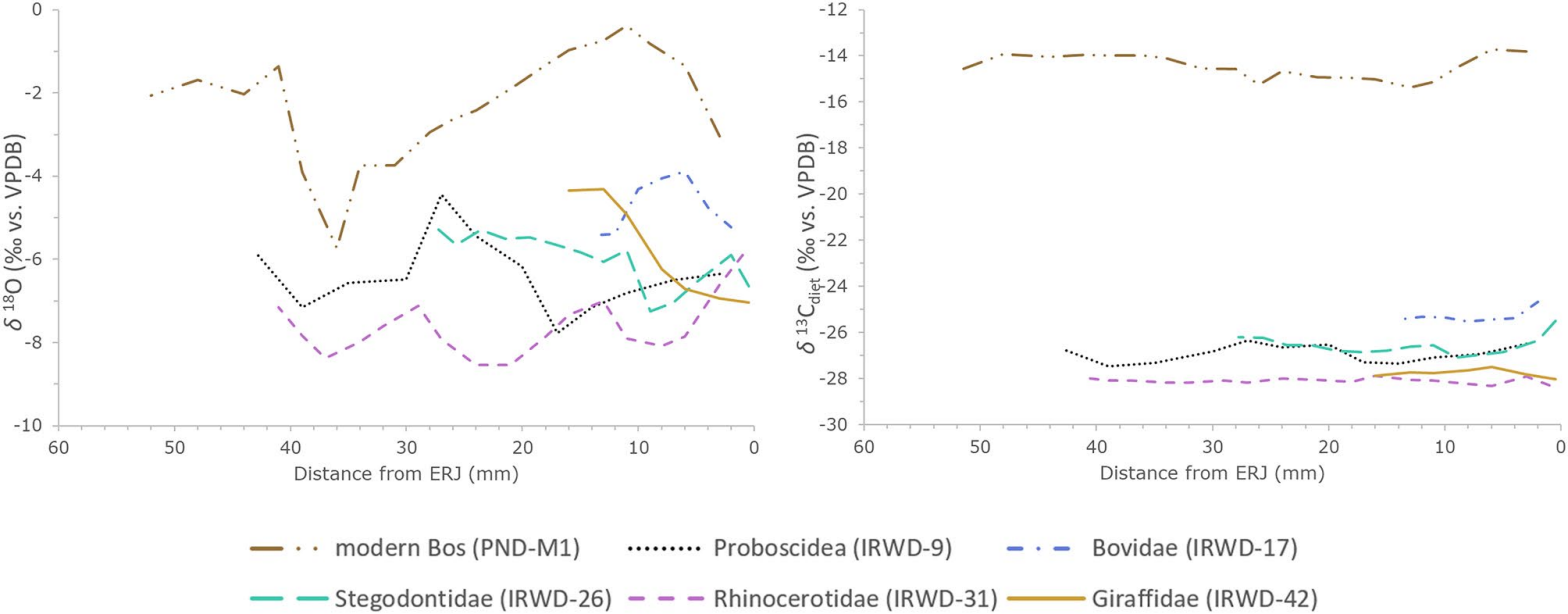
Comparison of the intra-individual serial sampling of a Miocene rhino from the Irrawaddy Fm. with a modern bovid from the Central Basin in Myanmar. Distance from ERJ (enamel root junction) is plotted in reverse order to correctly represent the enamel mineralization from oldest (left) to youngest (right).

For our data set, we used a modern bovid from Myanmar as reference for the seasonal variation of the *δ*^18^O values in the study area today. The *δ*^18^O values of the modern bovid from Myanmar are significantly higher than the Miocene values from the Yinseik locality (Table 1). We tested this with a Wilcoxon rank sum test comparing the modern with the Miocene bovid, which was the individual whose *δ*^18^O values were closest to the modern specimen (W = 6, *p*-value = 0.0001248). This indicates a wetter climate in Miocene Myanmar than today and is clearly visible when plotting the *δ*^18^O values against the distance from the ERJ (enamel root junction) (Fig. 3). A comparison of the minimum *δ*^18^O values from the Siwaliks and the Yinseik locality also shows that the climate in Miocene Myanmar was likely even wetter than in the contemporaneous Siwaliks (Fig. 2). Only the bovid from the Yinseik locality plots among the equids from the Siwaliks and are near the regression line. The specimen did not have a high tooth crown preserved, due to tooth wear, and therefore our data might not cover the time period of maximum seasonal precipitation, which would explain the higher minimum *δ*^18^O value in comparison to the other mammals from the same locality. An alternative explanation would be differences in metabolism. High *δ*^18^O values are expected in water independent species, because they obtain their water mostly from their food and not meteoric water, which reduces the loss of body water since it is recycled. However, modern bovids are not considered to be such a water independent species.

**Table 1:** Summary statistics for the intra-tooth serial sampling.

Specimen no.	No. of samples	Taxonomy	*δ*^18^O_VPDB_			*δ*^13^C_diet_			C&O covariance
			Min	Max	Amplitude	Min	Max	Amplitude	
IRWD-9	12	Proboscidea	− 7.3	− 4.1	3.2	− 27.2	− 26.2	1.1	0.2
IRWD-17	8	Bovidae	− 5.0	− 3.6	1.4	− 25.4	− 24.6	1.4	− 0.1
IRWD-26	14	Stegolophodon	− 6.8	− 5.5	1.2	− 27.2	− 25.4	1.8	0.1
IRWD-31	17	Rhinocerotidae	− 8.8	− 6.1	2.7	− 27.9	− 27.5	0.4	0.0
IRWD-42	7	Giraffidae	− 7.5	− 4.6	2.8	− 27.6	− 27.1	0.5	0.0
PND-M1	19	Bos	− 5.7	− 0.4	5.3	− 15.4	− 13.7	1.7	− 0.3

IRWD specimens are from the Miocene locality, whereas PND-M1 is a modern reference sample also originating from the Central Basin of Myanmar.

**Figure 4:**
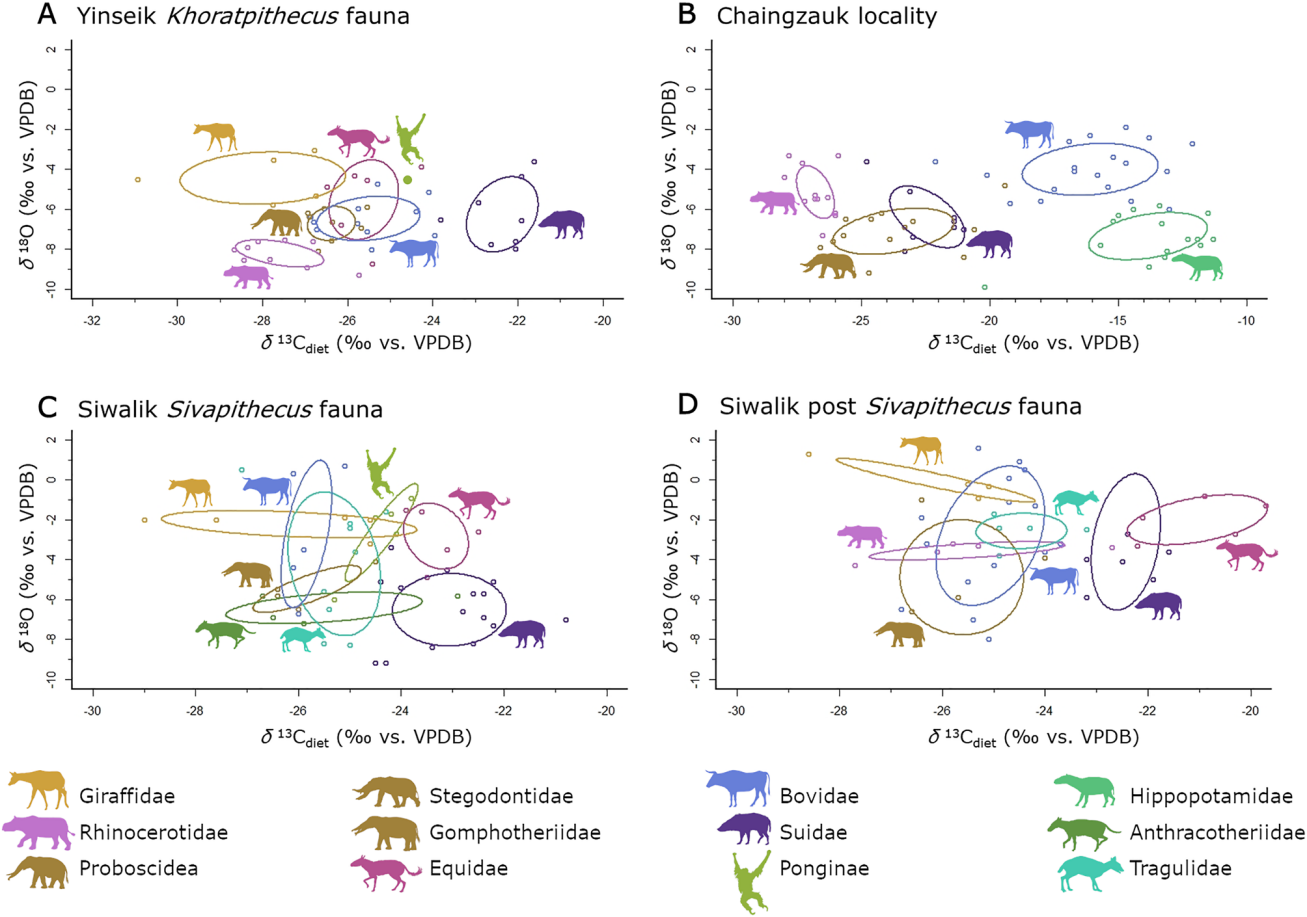
Bayesian niche modelling of four mammal communities. The ellipses mark the SEA (standard ellipse area) or core ecological niche, which corresponds to a 40% CI. Summary statistics are reported in Table SI 1. (**A**) Yinseik fauna including *K. ayeyarwadyensis* (~ 9.5 Ma), (**B**) Chaingzauk fauna (6 4 Ma)^25^, (**C**) Siwalik fauna including *Sivapithecus* (~ 9 Ma)^17^, (**D**) later Siwalik fauna (~ 8 Ma)^17^. Icons from PhyloPic and in public domain or under CC 3.0 license (Rhinocerotidae, Hippopotamidae, and Tragulidae by Zimices).

A Wilcoxon rank sum test showed that *δ*^18^O values of bulk samples from the *Khoratpithecus* fauna (Fig. 4a) are significantly lower in comparison with the *Sivapithecus* fauna (W = 4056, *p*-value = 1.245e−06) (Fig. 4c). This is also consistent with this interpretation. However, the amplitude of the *δ*^18^O values, defined as the difference between maximum and minimum values of one tooth, is smaller for the Miocene specimen than for the modern reference sample (Table 1). If we remove the outlier (PND-M1n) (tested with the Grubbs’ test for outliers (G = 2.5669, U = 0.6136, *p*-value = 0.0427)) of the modern bovid the amplitude drops from 5.3 to 3.5‰, which is still higher than the maximum amplitude from a Miocene specimen, the Giraffid (IRWD-42) with 2.8‰. This is consistent with a less pronounced difference in seasonal precipitation in the Miocene, as compared to the climate in modern day Myanmar.

In addition, we do not see the same pattern of covariation between the *δ*^13^C and the *δ*^18^O values. In the Miocene specimens, we see marginal positive correlations, whereas *δ*^13^C and *δ*^18^O values of the modern bovid are slightly negatively correlated. The stronger a positive or negative correlation is, the more seasonality effects an individuals diet, e.g. its quality or source. Hence, the results suggest a different influence of the seasonal extremes on the food resources in the Miocene than today. What the difference between a positive or negative covariation of *δ*^13^C and *δ*^18^O values reflects still needs to be characterised by further studies. The *δ*^13^C_diet_ values have been corrected for variations of isotopic composition of the CO_2_ in the atmosphere as well as isotopic enrichment factors from diet to enamel. A detailed discussion can be found in the Methods section.

### Paleoecology and niche partitioning of the Khoratpithecus mammal community

The *δ*^13^C_diet_ values of the *Khoratpithecus* fauna from the Irrawaddy Fm. at the Yinseik locality span almost the entire range of modern C_3_ plants (− 33 to − 22‰)^18^, with Giraffidae and Rhinocerotidae having the lowest and Suidae having the highest values (Fig. 4a). When we correct the *δ*^13^C values of C_3_ plants from the modern values reported by Bender^18^ to preindustrial ones (used in this paper to compare data sets from different eras to one another) the cut-off point for C_3_ vegetation lies at − 20.5‰. Higher values would indicate the presence of C_4_ plants in an individual's diet.

A comparison of the range of *δ*^13^C values of the Yinseik data set (− 30.3 to − 21.3‰) to the contemporaneous *Sivapithecus* fauna from the Dhok Pathan and Khaur regions of the Siwaliks (− 29.0 to − 20.8‰) shows that, both *K. ayeyarwadyensis* and *Sivapithecus* lived in a mosaic landscape. The isotopic data from the two areas are consistent with a woodland forest with open patches that were mostly occupied by suids. In the Siwalik fauna the equids also have *δ*^13^C values indicating more open habitats. In contrast, the equids from the Yinseik locality lived in more closed parts of the habitat according to their *δ*^13^C values. Therefore, it seems that the Yinseik habitat was a bit more densely forested than the Siwalik one. None of the mammals from the Yinseik locality have *δ*^13^C values above the cut-off point of − 20.5‰ including the equids, indicating a pure C_3_ vegetation. Considerably higher *δ*^18^O values of the *Sivapithecus* fauna (maximum = 0.7‰, mean = − 4.5‰) in comparison to the *Khoratpithecus* fauna (maximum = − 3.3‰, mean = − 6.5‰) are consistent with a slightly cooler and more humid climate at the Yinseik locality than in the Siwaliks. This pattern was also present in the comparison of minimum *δ*^18^O values discussed in the previous section (Fig. 2).

The *δ*^13^C value of *K. ayeyarwadyensis* place its habitat in the more open parts of the forest. It does plot among the data points with the highest *δ*^18^O values similar to those of giraffids and equids. For the former, consumption of leaves or fruit with higher *δ*^18^O values due to evapotranspiration is the most probable reason for these values whereas drinking from evaporated water sources like ponds seem more likely for the latter. As expected, the *δ*^18^O values of browsers foraging on leaves closer to the forest floor like the rhinos are lower than the ones from more arboreal browsers like giraffids.

In primates folivory/frugivory as well as vertical stratification in habitat use have been discussed as drivers of oxygen isotope fractionation^21,63^. Recent studies however showed that vertical stratification probably is the primary driver of variations of *δ*^18^O values^23,24^. Hence, the *δ*^18^O value of *K. ayeyarwadyensis* is consistent with foraging high up in the canopy. The corresponding *δ*^13^C value is also consistent with this interpretation, because the canopy effect is less pronounced high up in the canopy leading to higher *δ*^13^C values of animals foraging there. A predominantly frugivorous diet, without any evidence for the consumption of hard objects has been reconstructed using dental microwear texture analysis and dental topography of *K. piriyai* and *K. chiangmuanensis*, the two closest allies of the Myanmar species^64^, a subsistence strategy consistent with our data. *K. piriyai* is known from fossil localities in Thailand contemporaneous to the Yinseik locality with *K. ayeyarwadyensis* discussed in this paper where it is associated with a similar mammal fauna^6,7^.

The two younger data sets from the Chaingzauk (6–4 Ma) locality in Myanmar (Fig. 4b) and the post-*Sivapithecus* (~ 8 Ma) layers from the Siwaliks (Fig. 4d) show similar trends. A slight shift towards more positive *δ*^13^C values in the Siwaliks (− 28.6 to − 19.7‰) and a drastic shift plus an increasing range for *δ*^13^C values in the Chaingzauk data (− 28.0 to − 11.3‰) both illustrate an ongoing opening of the landscape. As the Chaingzauk locality is 2–4 Ma younger than the post-*Sivapithecus* Siwalik data set, it becomes apparent, that the opening of the landscape was an ongoing process in South and Southeast Asia throughout the Late Miocene. The introduction of C_4_ plants results in the adaptation of the ecological niches of some groups of mammals, especially the suids and equids in the Siwalik and bovids and rhinos in the Chaingzauk community.

### Assessing the ecological niches of fossil pongines

**Figure 5:**
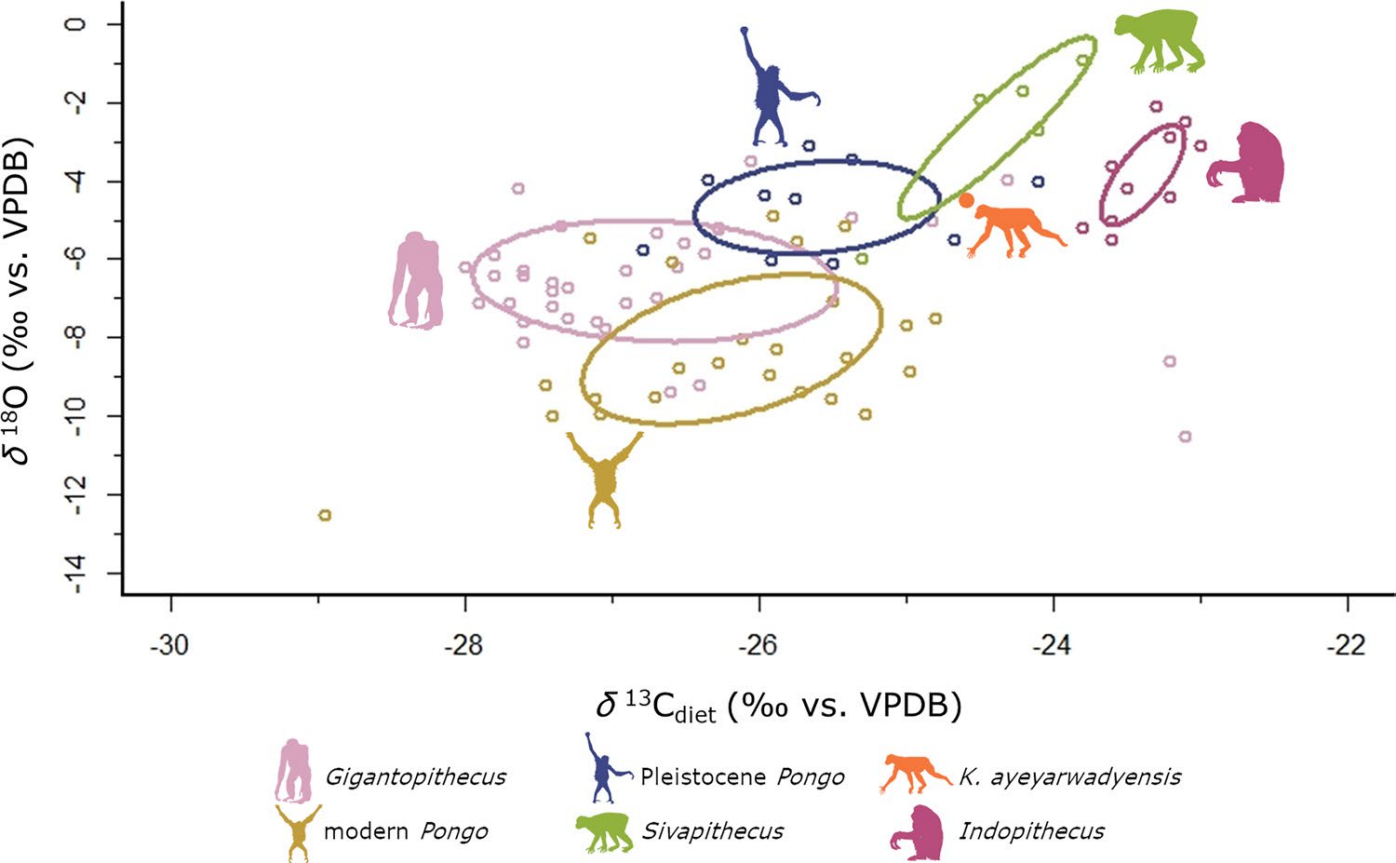
Bayesian niche modelling of fossil and extant pongines. The lines encompass the standard ellipse area or core ecological niche, corresponding to a confidence interval of 40%^60^. Icons are public domain (*Gigantopithecus*, *Indopithecus*) or Creative Commons 3.0 license [modern and Pleistocene *Pongo* by Gareth Monder, *Khoratpithecus* by Mateus Zica (modified by T. Michael Keesey), and *Sivapithecus* by Nobu Tamura (modified by T. Michael Keesey)] via PhyloPic.

The ecological niches of *Sivapithecus* and *K. ayeyarwadyensis* inferred from the modelled isotopic niches and the comparison with associated mammal fauna look very similar to each other and to modern orangutans in some general characteristics. The ecological niches of the fossil pongines are consistent with predominantely frugivorous, arboreal forest dwellers, characteristics that can also be applied to orang-utans today. The direct comparison of the ecological niches of the various fossil and extant pongines however, indicates differences in their ecology and habitat use (Fig. 5). The facts that only one sample of *Khoratpithecus* was available and we do see a high variation in both the *δ*^13^C and *δ*^18^O values of the other pongine genera do limit the interpretations we can make in regard to this specific pongine. We can however give a first approximation of its ecology and show that it is consistent with the trends visible through the contemporaneous *Sivapithecus*, for which the sample size (n = 5) was large enough to model an isotopic niche with reasonable confidence^60^.

Modern species of *Pongo* are the most frugivorous of all extant apes. Although the diets of the two Miocene pongines were reconstructed as predominantly frugivorous, the addition of some hard objects like seeds and nuts for *Sivapithecus* but not for *Khoratpithecus*^64–66^, highlights a difference in the dietary ecologies between the genera. Modern orangutans rely on a number of fallback foods (e.g. inner bark, pith, young leaves, and flowers)^67,68^ in times of fruit scarcity, which are tough, but not hard, and have therefore different microwear patterns^69^.

To test the similarity of habitat between *Sivapithecus* and Pleistocene *Pongo*, we conducted a Wilcoxon Rank Sum test to see if there is a difference in *δ*^13^C_diet_ values between both pongines. The result shows a significant difference (W = 4.5, *p*-value = 0.01422). Therefore, although the ecological niche of *Sivapithecus* and *K. ayeyarwadyensis* within their respective mammal communities resemble that of modern *Pongo*, a comparison of these ecological variables with the other pongines on a broader scope (Fig. 5) reveals that the microhabitats they occupied or their habitat use differed significantly.

Although all groups of pongines lived in a forested habitat, *K. ayeyarwadyensis*, *Sivapithecus* and *Indopithecus* seem to be located in a more fragmented forest or higher up in the canopy where the canopy effect is less pronounced. The ecological niches of both modern and Pleistocene *Pongo* as well as *Gigantopithecus* are indicating a more closed canopy or foraging lower down in the canopy or even on the ground where the canopy effect is more pronounced. A difference in forest structure between the Miocene and Pleistocene is also suggested by palynological data, paleosol isotopic data, and climate and vegetation models^52,70–75^. The change in vegetation and landscape structure could be explained by the marked climate change during the end of the Neogene^52^.

The difference in isotopic niches between Miocene and the Pleistocene pongines as well as modern *Pongo* is likely not only related to the general structure of the habitats, but also to the nature of habitat use. The data on the Miocene pongines *K. ayeyarwadyensis* and *Sivapithecus* are consistent with using areas higher up in the canopy, as they have both higher *δ*^18^O and *δ*^13^C_diet_ values. This correlates nicely with a lower body mass in comparison with the Pleistocene pongines. However, the estimated body mass of around 150 kg or more of *Indopithecus*, comparable with extant *Gorilla*^76^, is more similar to *Gigantopithecus*^77^ and makes an arboreal habitat less probable. Nevertheless, its isotopic niche is more similar to the Miocene pongines from Myanmar and Pakistan than to *Gigantopithecus* or *Pongo*. As the similar sized gorillas exploit arboreal resources and climb trees, a similar behaviour of *Indopithecus* would be possible. Alternatively, its isotopic niche would be also consistent with a more open habitat, which has already been suggested for *Indopithecus*^78^. In addition, the interpretation of the *Indopithecus* ecological core niche based on the data available for this study should be regarded as preliminary, especially for the *δ*^18^O values, given the fact that all the data come from one individual from which intra-individual serial samples were taken using laser ablation. Although we used the *δ*^18^O values corrected by an offset of 5.1‰ (“CO_3_ equivalent”)^26^ for our study, we cannot exclude that they are not biased due to the different sampling methodology.

*Gigantopithecus* seems to have occupied the most densely-forested habitats, given the low *δ*^18^O and *δ*^13^C_diet_ values. Within this genus, there are two outliers with high *δ*^13^C values, which are two of the samples from the Early Pleistocene Longgudong Cave in Southern China^28^. These values are consistent with a more open habitat for these two individuals, although their *δ*^18^O values do not correspond to plants subjected to evaporative stress as food sources. However, Jiang and collaborators re-analysed one of these two samples and reported a value 1.6‰ lower^79^. It cannot be ruled out that the variation is partly caused by the different pretreatment method applied by Nelson^28^.

Wilcoxon Rank Sum test confirmed that Pleistocene and modern *Pongo* did not differ significantly in their *δ*^13^C values (W = 86.5, *p*-value = 0.2123). Hence, modern orangutans have not yet been pushed by human pressure into denser forest habitats or to the forest margins and more open spaces due to deforestation around a century ago, which is the time period our sample dates to. All the dates are reported in Table SI 2 and Table SI 3. These results also show that although their habitats became increasingly fragmented, pongines remained dependent on closed habitats and were unable to adapt and move into more open forested woodlands.

The data are consistent with shifts in *δ*^18^O consistent with differences in vertical stratification^23,24^. Decreasing *δ*^13^C_diet_ values from the Miocene pongines (except in *Indopithecus*), to Pleistocene and modern *Pongo*, to *Giganthopithecus* are also consistent with this interpretation, as the canopy effect is more pronounced lower down in the canopy. This seems plausible for *K. ayeyarwadyensis*, *Sivapithecus*, Pleistocene *Pongo* and *Gigantopithecus* as this coincides nicely with increasing body mass in these three genera. The higher body mass of *Pongo* in comparison to both *K. ayeyarwadyensis* and *Sivapithecus* would make foraging high up in the canopy and close to terminal branches increasingly difficult. However, brachiation in conjunction with high hip flexibility in *Pongo* counters the negative impact of higher body mass during climbing to a certain extent, due to weight distribution over many smaller branches^80–82^.

At first it sound promising to relate the decreasing *δ*^18^O values from the Miocene to the Pleistocene genera (see in Fig. 5) to the increasing competition with cercopithecoid monkeys, which became increasingly abundant in Southeast Asia from the Late Miocene and especially the Plio-Pleistocene onward^83–85^,. Nevertheless, the higher body mass of pongines in comparison with the contemporaneous monkeys makes it very unlikely that the former had to resort to lower canopy layers for foraging due to increased competition^86^. On the contrary, it has been suggested that monkeys that are capable of eating unripe fruit create a pattern of ripe fruit availability that is more restricted to terminal branches and therefore forcing apes to adapt to exploit these resources^80^.

As discussed earlier on, it was necessary to assess to what extent changes in *δ*^18^O values might reflect climatic changes over time and geographical distance. Although climatic changes were responsible for part of the *δ*^18^O variation that we see between the different pongine genera (Fig. 5), we are confident that an ecological signal is also visible. Especially between the Miocene and Pleistocene taxa, the relative position of *δ*^18^O values of bovids, pongines and suids across time and space (see more detailed discussion in the Supplementary Information) and the overall consistency of our interpretations with body mass differences between the genera. The data are therefore consistent with the possiblity that there is a difference in the usage of the canopy between the two Miocene pongines *Sivapithecus* and *K. ayeyarwadyensis*, which could forage even higher up, and *Pongo*. Relative position of *δ*^18^O values of bovids, pongines and suids suggest that the difference between Pleistocene and extant *Pongo* however is probably predominantly caused by differences in climate between the islands of the Sunda Shelf and mainland Southeast Asia. This is consistent with the *δ*^18^O values of modern *Pongo* being well correlated well with *δ*^18^O values of precipitation from Sumatra and Borneo. These values are lower on the islands in the Sunda Shelf as compared to mainland Southeast Asia, predominantly caused by a more intense monsoon^87^. These preliminary interpretations should be further investigated with bigger data sets for a more robust assessment of the variations of *δ*^18^O values as they span a large spatio-temporal range and variation in these values can be caused by many different factors^22^.

## Conclusions

*Khoratpithecus ayeyarwadyensis*, a Late Miocene pongine from Myanmar, lived in a woodland forest. The *δ*^13^C value is consistent with a microhabitat where the canopy effect is not strongly pronounced; so either in the lighter forest or higher up in the canopy, but not in the dense understory forest. Its high *δ*^18^O value is similar to those of sympatric giraffids and equids. Hence, the data are consistent with *K. ayeyarwadyensis* foraging higher up in the canopy where evapotranspiration leads to a depletion of ^18^O in food and water sources. Given that we could only sample one specimen of this genus, this only gives a first indication of the palaeoecology of *K. ayeyarwadyensis*, and should more specimen of this important pongine genus become available for stable isotope analysis, it would further improve our understanding of its palaeoecology and make our interpretations more robust. The precipitation regime in the habitat of *K. ayeyarwadyensis* was similar to the monsoon regime in present-day Myanmar, although seasonality was probably less pronounced. The high *δ*^18^O value is consistent with a diet consisting of plant tissues enriched in the heavier ^18^O isotope due to evapotranspiration like fruit or leaves. However, the results from dental microwear texture analysis of *K. piriyai* from Thailand suggest a predominantly frugivorous diet without the use of tough objects, so *K. ayeyarwadyensis* was likely also a frugivore and not a folivore. Overall, the habitats of these two Miocene hominoids seem to be similar to that of the modern *Pongo*, however the exploitation of these habitats and the ecological niches occupied by each species seem to be distinct. In the case of *K. ayeyarwadyensis* and *Pongo* they might use similar resources, but at different levels in the canopy.

The comparison of all Asian Miocene apes showed that the reconstructed ecological niches of *Sivapithecus* and *K. ayeyarwadyensis* seem to be superficially similar to the modern *Pongo*. They are all three predominantly frugivorous primates foraging high up in the canopy of forests with a seasonal monsoon-like precipitation regime. However, the density of the Miocene forest in the Siwalik and Irrawaddy region seems to be more fragmented with more deciduous vegetation. The overall climate was also drier than in modern Indonesia. Palynological and isotopic data on paleosols and benthic foraminifera as well as climate models suggest a similar development in vegetation structure and climate^52,70–75^. *Gigantopithecus* was adapted to foraging in denser forests, and given its immense body mass this was likely the the understory or forest floors. On the other hand, the data from modern and Pleistocene *Pongo* are consistent with using resources in the lower areas of the forest canopy.

As we compare pongine genera from different time periods with one another it was necessary to correct the *δ*^13^C values for changes in the isotopic composition of the atmospheric CO_2_. Such a correction is not possible for the *δ*^18^O values, as their variation is impacted by many different factors. We therefore assessed if some of the variation can be attributed to changes in ecology. We do however acknowledge, that the interpretations of variations and shifts in *δ*^18^O values are less robust, than the ones based on the *δ*^13^C values. Large scale investigations of *δ*^18^O variation over the spatio-temporal range of this study would improve the robustness of the interpretations.

As mentioned in the introduction, the present study contributes to improving our understanding of the evolutionary ecology of the fossil pongines that can provide valuable insight for current orangutan conservation. It seems that at least at the time from which the samples of modern *Pongo* originate, around a century ago, there was still enough suitable habitat available for the orangutans not to be pressured to leave their ancestral niche. However, the shifts in the environment we see in the isotopic niche modelling of the *Sivapithecus* and the associated *K. ayeyarwadyensis* fauna to later time periods after the decline and extinction of these Miocene ponginesillustrate the fate, which awaits the Southeast Asian apes if the habitats become more and more fragmented that of extinction.

## Supplementary Information

Supplementary Information.

Supplementary Table 1.

Supplementary Table 2.

Supplementary Table 3.

Supplementary Table 4.

## Data Availability

All data generated or analysed during this study are included in this published article (and its supplementary information files).
