# Supplementary material for: Evolutionary ecology of Miocene hominoid primates in Southeast Asia
Source: Sci Rep. 2022 Jul 12;12:11841. doi: 10.1038/s41598-022-15574-z (PMC9276763; doi:10.1038/s41598-022-15574-z)
Supplement: Supplementary file 1 — Supplementary Information. [file 41598_2022_15574_MOESM1_ESM.docx]

# Supplementary Information

## Pairwise comparison of *δ*^18^O values of pongines, suids and bovids

To facilitate a more accurate interpretation of the differences in *δ*^18^O values we systematically compared the pongine values with those of bovids and suids. With this method we tried to estimate how much of the variation was a result of climate and temperature variations over time and what can be actually attributed to changes in the ecology of the fossil and modern pongines. Bovids and suids were chosen, because we have data from these taxonomic groups associated with all the pongines. The data used for this comparison are reported in Table SI 3.

At first, we explored how the *δ*^18^O values vary within the taxonomic groups over the different geological eras (Fig. SI 1). Bovids and pongines show a very similar pattern of *δ*^18^O variation with the exception of the Holocene where pongines show lower values. However, especially in the Early Pleistocene, from where most of the *Pongo* and *Gigantopithecus* samples are, there is much more variability in the pongines, as they continuously cover the whole range of *δ*^18^O values. This is a first slight indication that there is the potential of interpreting the *δ*^18^O in terms of differences in the ecological niches of fossil pongines. The suids do not show any variation in *δ*^18^O except for lower values in the Holocene.

In both the pongines and the suids the Holocene values are lower than in the other eras. This can be attributed to the fact that all of the samples come from islands on the Sunda Shelf, whereas the data set for the Holocene bovids encompasses sites from the Southeast Asian mainland. Lower *δ*^18^O values for the Sunda Shelf region were expected given the higher monsoon intensity there in comparison to mainland Southeast Asia. This is also confirmed by climate and vegetation models^35,36,47^.


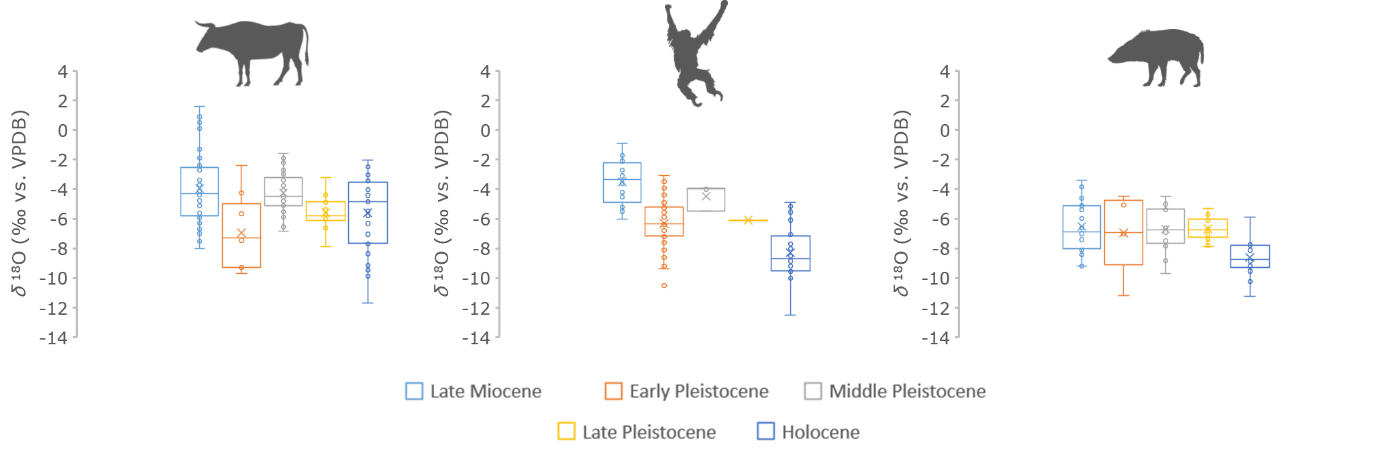


Fig. SI 1 Box plot of the *δ*^18^O values of the three taxonomic groups (bovids – left, pongines – middle, suids – right) per geological era. Icons obtained via PhyloPic and in Public Domain.

In a second step, we plotted each fossil pongine next to the associated bovids and suids (Fig. SI 2). Although the relative positions of the pongines to the other two groups varies from genus to genus, bovids always have higher *δ*^18^O values than suids. This is an indication that the differences in *δ*^18^O values in fossil pongines has some significance for the specific ecological niches of the different genera and does not only reflect climatic changes over time.


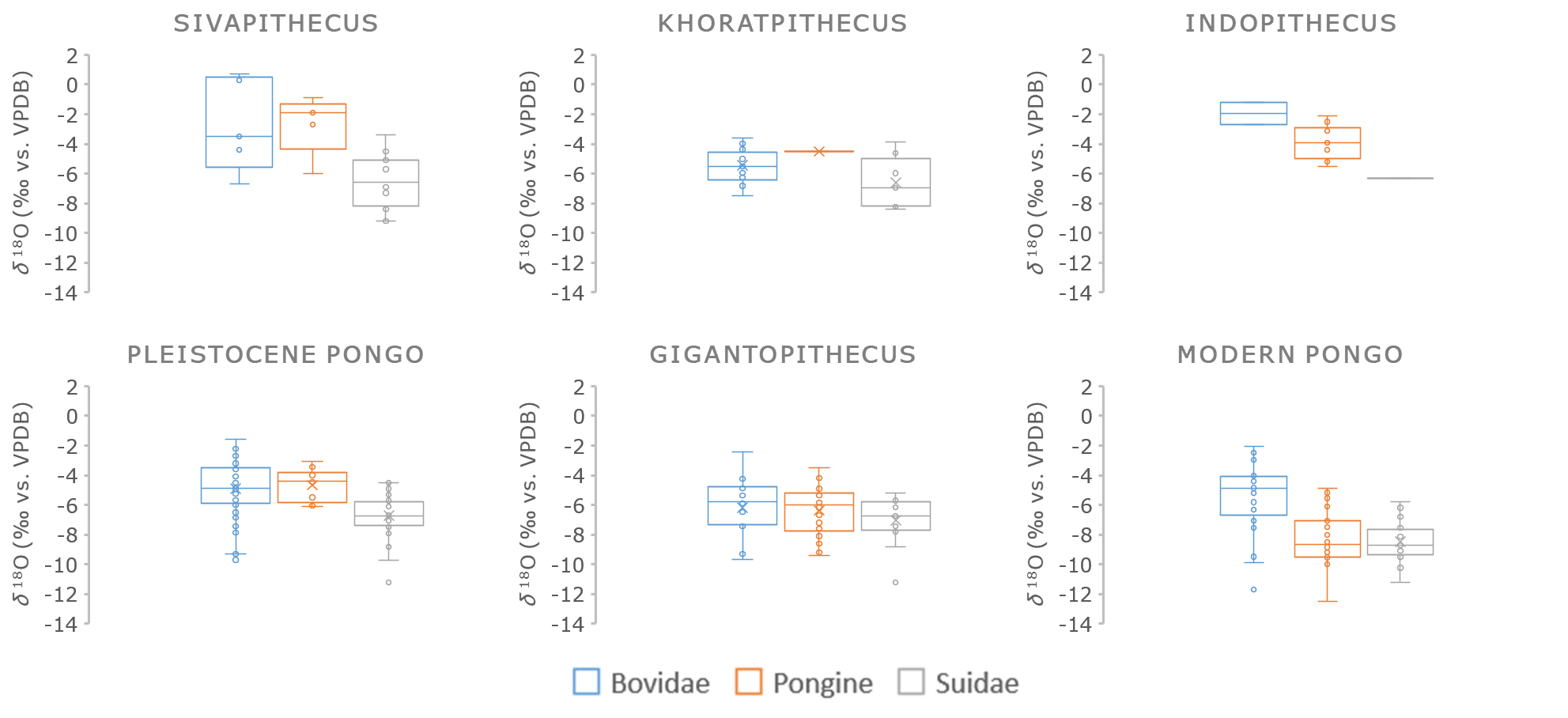


Fig. SI 2 Box plot of the *δ*^18^O values of fossil and modern pongines with the associated bovids and suids.

## Calculation of niche overlaps

Based on the isotopic niche modelling in SIBER we quantified the niche overlap based on the SEA_C_, the standard ellipse area corrected for small sample sizes that is visualized in the plots in Fig. 4 and 5 using R. It is possible to calculate three different percentages for each overlapping instance, where A_O_ is the overlap area of the two SEA_C_s:

- The percentage of niche area of taxon A (A_A_) that overlaps the niche space of taxon B (A_O_/A_A_)
- The percentage of niche area of taxon B (A_B_) that overlaps the niche space of taxon A (A_O_/A_B_)
- The percentage of the total niche space of the two taxa that is shared between them, which is calculated with the following formula:

$\% overlap = \frac{A_{O}}{A_{A}+ A_{B}- A_{O}} \times100$ ^71^ (SI 1)

For the comparison of niche overlap between the different taxonomic groups and pongine genera we focused on the percentage of the total niche space, that is shared between the compared groups.

## Niche overlap for the *Khoratpithecus* and *Sivapithecus* mammal faunas


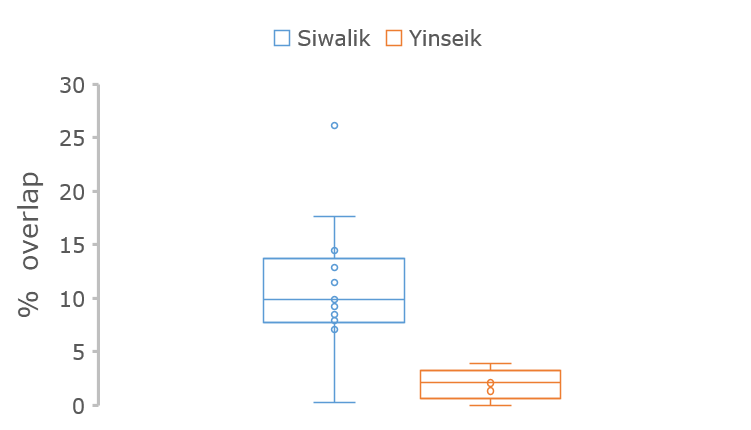
To compare the structure of niche partitioning between the *Sivapithecus* and *Khoratpithecus* mammal fauna we calculated the % overlap between all the groups.

A Wilcox rank sum test revealed, that the distributions are not significantly different
(W = 273.5, p-value = 0.06895), when all the possible pairings between two taxonomic groups where the % overlap is 0 % are included. Hence, the isotopic niche modelling does not indicate a significantly heightened pressure due to competition in one of the two mammal communities. This would be consistent with an interpretation of the difference in dietary preference between the two fossil pongine genera based on dental microwear as being related to ecology and not to competition pressure.
If we do exclude all instances where the % overlap is zero (Fig. SI 3) the difference is significant (W = 48, p-value = 0.01008). According to this result, here is a difference in competition potential between the mammal community of *Sivapithecus* and *Khoratpithecus.* That could be an explanation of the incorporation of harder objects in the *Sivapithecus* diet. However, difference in the number of taxonomic groups present between the two datasets definitely has an impact on the statistical tests applied.

Fig. SI 3 Box plot of the Sivapithecus (Siwalik, left) and Khoratpithecus (Yinseik, right) mammal faunas comparing the niche overlap of the taxonomic groups (% overlap). Groups with an overlap of 0 % were not included in this plot.

Including all the possible pairings between two taxonomic groups where the % overlap is 0 % resulting in mean values of 5.1 % for the *Sivapithecus* fauna and 2.4 % for the *Khoratpithecus* fauna. When excluded the mean % overlap shifts to 5.1 % for *Sivapithecus* and 0.8 % for the *Khoratpithecus* fauna. For the visualisation of the distribution of % overlap between the two communities we excluded these groups. A_O_ values and % overlap for each pairing is reported in Table SI 4.

## Niche overlap between fossil and extant pongines

*Gigantopithecus* (G) and modern *Pongo* (mP) (A_O G mP_ = 1.6) had the biggest niche overlap between the fossil and extant pongines (16.46 %), which is already clearly visible in Fig. 4, followed by *Sivapithecus* (S) and Pleistocene *Pongo* (PP) (A_O S PP_ = 0.18) (3.91 %) and *Gigantopithecus* and Pleistocene *Pongo* (A_O G PP_ = 0.2) (2.3 %). The most likely reason for the niche overlap between *Gigantopithecus* and modern *Pongo* not being bigger, is due to the bias introduced by the impact of climate differences between the Holocene Sunda Shelf and Pleistocene mainland Southeast Asia. This has been discussed in the chapter on the pairwise comparison of *δ*^18^O values of pongine, bovid, and suid isotopic data. Given that we could only sample one specimen of *K. ayeyarwadyensis*, no Bayesian standard ellipse could be modelled for this taxon.

# Supplementary Material


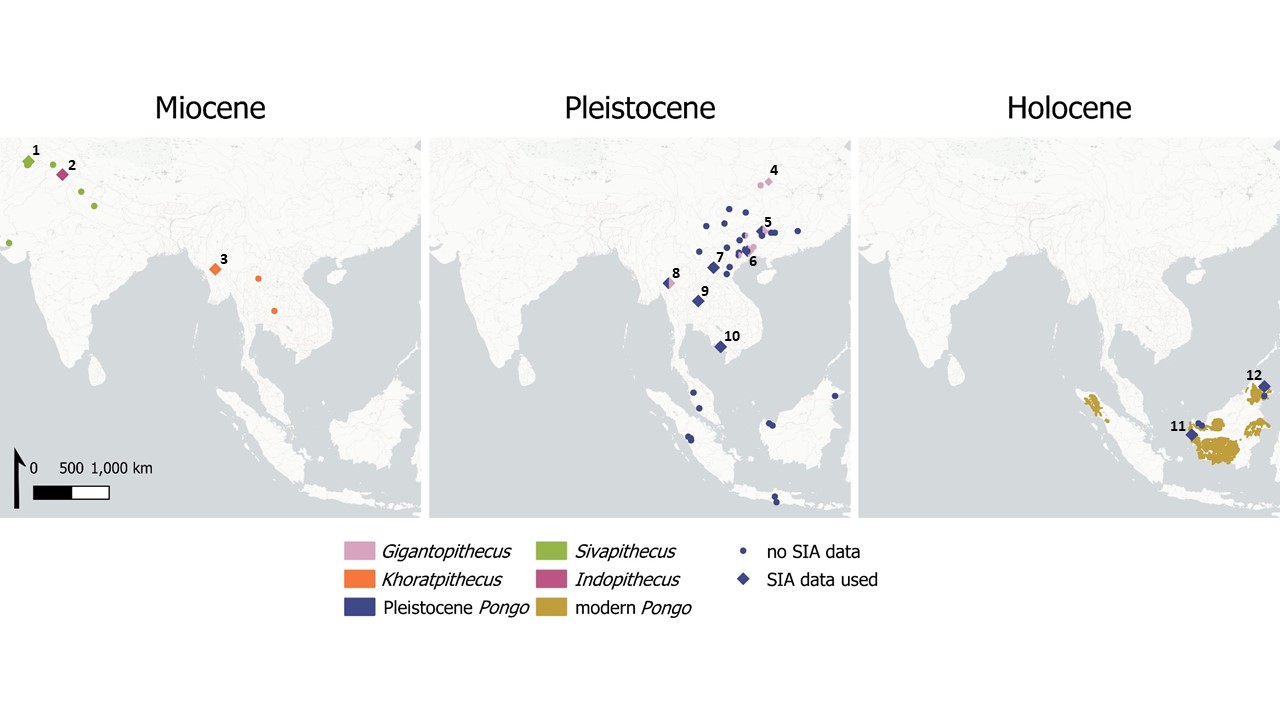


Fig. SI 4 Maps of fossil pongine localities from the Miocene to the Holocene including the current distribution. Sites from which stable isotope data was used in this study: 1 Dhok Parthan, Siwaliks, Pakistan; 2 Haritalyangar, Siwaliks, India; 3 Yinseik, Irrawaddy Fm., Myanmar; 4 Longgudong, Jianshi, China; 5 Juyuandong, Liucheng Cave, Guangxi, China; 6 Sanhe Cave, Guangxi, China; 7 Nam Lot, Laos; 8 Pha Bong, Thailand; 9 Thum Wiman Nakin, Thailand; 10 Boh Dambang, Cambodia; 11 Pontianak, Indonesia; 12 Sandakan, Malaysia. The map was created using QGIS 3.16.

Table SI 1 Summary statistics of the four late Miocene mammal communities we compared to get information on the palaeoecology of *K. ayeyarwadyensis* as well as the fossil to extant pongines used in the assessment of ecological continuity. TA refers to the convex hull area and SEA_C_ for the area of the modelled standard ellipses corrected for small sample sizes^17^.

Table SI 2 This table reports all the raw SIA data including metadata from the Miocene mammal faunas (including *Khoratpithecus* and *Sivapithecus*) from the Yinseik and Chaingzauk localities in Myanmar, the *Sivapithecus* and post-*Sivapithecus* horizons form the Siwaliks of Pakistan, as well as the modern bovid from Myanmar. We included both the raw data (*δ*^13^C_VPDB_) as well as the corrections applied to each data point and the corrected values (*δ*^13^C_diet_) used for our analysis. *δ*^18^O_SMOW_ values were calculated using the formula proposed by Coplen^72^.

Table SI 3 This table reports all the raw SIA data used in this study, including metadata from fossil and modern pongines as well as data from bovids and suids used for the pairwise comparison with the pongines ranging from the Miocene to the Holocene. We included both the raw data (*δ*^13^C_VPDB_) as well as the corrections applied to each data point and the corrected values (*δ*^13^C_diet_) used for our analysis. *δ*^18^O_SMOW_ values were calculated using the formula proposed by Coplen^72^.

Table SI 4 Summary of A_0_ and the % overlap per taxonomic group for the mammal faunas from the Yinseik locality (*Khoratpithecus* fauna) and the Siwaliks (*Sivapithecus* fauna) used in the comparison of niche overlaps.
